# A review of published literature on emergency medicine training programs in low- and middle-income countries

**DOI:** 10.1186/1865-1380-6-26

**Published:** 2013-07-17

**Authors:** Anna K Nowacki, Megan Landes, Aklilu Azazh, Lisa M Puchalski Ritchie

**Affiliations:** 1 Department of Medicine, Division of Emergency Medicine, University of Toronto, Toronto, ON, Canada; 2 Department of Family and Community Medicine, Division of Emergency Medicine, University of Toronto, Toronto, ON, Canada; 3 University Health Network, Toronto, ON, Canada; 4 Department of Emergency Medicine, College of Health Sciences, Addis Ababa University, P. O. Box 9086, Addis Ababa, Ethiopia; 5 Department of Emergency Medicine, University Health Network, 200 Elizabeth Street, Toronto, ON M5G 2C4, Canada

**Keywords:** Emergency medicine, Residency, Training programs, Education

## Abstract

**Background:**

The objective of this review is to identify and critically evaluate the published literature on emergency medicine (EM) training programs in resource-limited health-care settings in order to provide insight for developing EM training programs in such health systems.

**Methods:**

A literature search was conducted up to the end of April 2011 using MEDLINE, EMBASE, The Cochrane Library, EBM Reviews, Healthstar and Web of Science databases, using the following search terms: Emergency Medicine, Emergency Medicine Services, Education Training Residency Programs, Emergency Medical Systems and Medical Education, without limitation to income countries as outlined in the World Bank World Trade Indicators classification 2009-2010 (World Trade Indicators Country Classification by Region and Income, July 2009-July 2010). As the intent of the review was to identify and critically evaluate the literature readily available (published) to LMICs developing EM programs, the gray literature was not searched.

**Results:**

The search yielded 16 articles that met the final inclusion criteria. As the majority of articles provide a narrative description of the processes and building blocks used in developing the residency programs reported, we present our results in narrative format. By providing a summary of the lessons learned to date, we hope to provide a useful starting point for other resource-limited settings interested in establishing emergency medicine specialty training programs and hope to encourage further information exchange on this matter.

**Conclusions:**

The results of the review indicate that EM training is in its infancy in resource-constrained health-care systems. There are few detailed reports of these programs successes and limitations, including efforts to optimize graduate retention. Despite the paucity of currently published data on the development of EM residency training programs in these settings, this review demonstrates the need for encouraging further information exchange to aid in such efforts, and the authors make specific recommendations to help guide future authors on reporting on such efforts.

## Background

Emergency medicine (EM) is a relatively new specialty around the world, having been officially recognized in both Europe and North America only in the latter half of the twentieth century. To this day, emergency departments (EDs) in many countries are not staffed with specialists with specific training in the discipline, but rather with rotating off-service staff physicians or with residents and interns. This is particularly true in resource-limited settings where there are relatively few EM trained staff, few or no EM training programs, and limited organization of emergency medicine services. Further increasing the burden on weak EM services in these health-care settings is the frequent lack of access to primary care, leading many patients to seek delayed treatment, often in an acute or critical state. As a result, resource-limited settings experience a significant mismatch of needs and services: high rates of critically ill patients and constrained or underdeveloped EM systems. The need for EM services in such settings is clear, yet to date efforts toward establishing EM in resource-limited settings have been slow. In Ethiopia, initiatives are supported by the Ministry of Health to increase emergency medicine capacity, and in particular the number of trained EM professionals. Ethiopia, like much of the region, continues to suffer from an ongoing ‘brain drain’ [1], with many Ethiopian-trained physicians pursuing specialty-training and employment abroad. As part of an effort to combat this, in 2010 Addis Ababa University initiated the first emergency medicine residency training program at Tikur Anbessa Hospital. The first cohorts have entered a 3-year training program and are expected to become the next generation of teachers and leaders of EM in Ethiopia. As part of a group of emergency medicine physicians and residents at the University of Toronto, we have been collaborating in the above-mentioned endeavor.

### Importance

To inform the effort at Addis Ababa University in the establishment of Ethiopia’s first EM residency training program, we conducted a systematic review of the published literature on emergency medicine training programs in resource-limited health-care settings. We focus on low- and middle-income countries (LMICs) in particular, given the unique challenges facing resource-constrained settings in developing EM as compared to high-income countries. These challenges include: limited access to health care for the general population, limited resources within the health-care system, as well as an emerging double burden of both communicable and non-communicable diseases [2].

### Goals

To our knowledge, to date there has been no such review, and we aim to provide a useful resource for other LMICs interested in developing EM residency training programs, as well as for those interested in collaborating with these groups.

## Methods

### Search strategy

We searched the following electronic databases for relevant articles, without restriction to language, participant age or study design:

•Ovid MEDLINE(R) In-Process & Other Non-Indexed Citations <April 27, 2011>

•Ovid MEDLINE(R) <1948 to April week 3 2011>

•EMBASE <1980 to 2011 Week 16>

•EBM Reviews - Cochrane Database of Systematic Reviews <2005 to April 2011>

•EBM Reviews - Database of Abstracts of Reviews of Effects <2nd Quarter 2011>

•OVID HEALTHSTAR <1966 to March 2011>

•Citation Databases: Web of Science searched April 28, 2011

The literature search was conducted using the following search terms: Emergency Medicine, Emergency Medicine Services, Education, Training, Residency Programs, Emergency Medical Systems and Medical Education. The results were then limited to low, low-middle, and upper-middle income, excluding high-income countries based on the World Bank World Trade Indicators classification 2009-2010 – [3]. The full search strategy for each of the databases may be obtained from the authors upon request. As an example, the full EMBASE search strategy is demonstrated in Additional file 1. A search of the gray literature was not undertaken as one of our goals was to identify gaps in currently published literature in this area potentially resulting in recommendations for future publications.

### Inclusion/exclusion criteria

Studies that were included met the following criteria:

(1) Described an emergency medicine, teaching and/or training program(s)

(2) Programs focused on training physicians or physician trainees at any level, from medical student to continuing medical education modules (CMEs). (Given that many resource-poor countries complete GP training first and often work for a period of time before returning for specialty training, CMEs were included, to avoid excluding a significant group of trainees)

(3) Described training programs in low- and/or middle-income countries. The search initially included only low- and low-middle-income countries, but was later extended to include all middle-income countries, given the similar lessons in and challenges of attempting to establish EM training programs in many of these settings.

Exclusion criteria were as follows:

(1) Articles reporting a case report or series

(2) Articles representing a general topic review, with the exception of reviews of EM training programs

(3) Articles reporting training programs for non-physicians.

In addition, the search resulted in a large number of articles on specific topics of potential relevance to EM, including: disaster preparedness and/or complex humanitarian responses, emergency obstetrics and emergency contraception and toxicology. As many of these were general topic reviews, they were excluded unless they described a course or training program designed for or part of an emergency medicine training program for physicians.

#### Selection process

**Figure 1:**
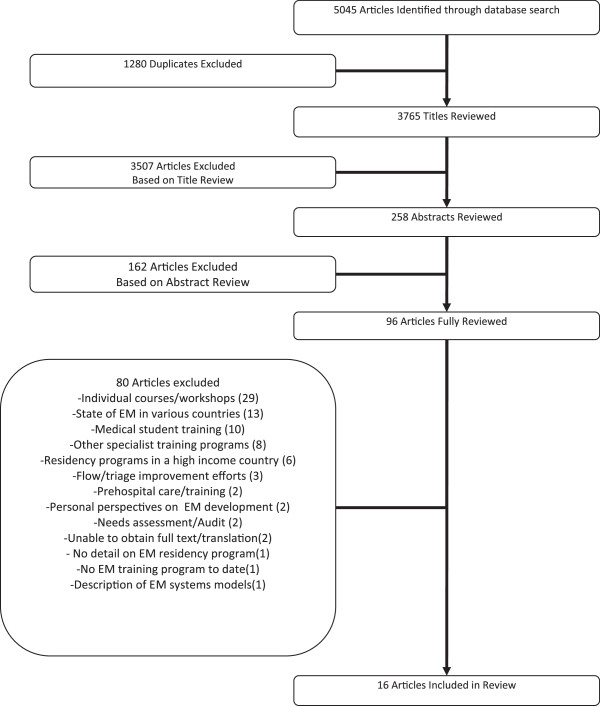
Schematic of study selection process.

Studies were selected as described below. The selection process consisted of three steps: a title review, abstract review and full article review. Articles were independently reviewed by three reviewers at each stage, with final selection for inclusion at each stage based on discussion and group consensus. The search returned 5,045 titles. After the removal of duplicates, 3,765 titles were reviewed, from which 258 abstracts were chosen for review and 96 chosen for full article review. As we were unable to obtain the full text of 1 article [4], 95 articles were reviewed in full, with 16 meeting the full inclusion criteria (see Figure 1).

## Results

Sixteen articles fulfilled the inclusion criteria (see Table 1). The majority (15) were review articles or updates, providing an overview of emergency medicine in 14 LMICs, as well as descriptions of EM training programs. The articles had broad geographical scope spanning 14 countries on 5 continents.

**Table 1:** Article summary

**Author**	**Country**	**Focus of article**	**Curriculum**	**Duration/ format**	**Partnerships/ exchanges**	**Start date of program**
Abbadi et al.	Jordan	Describes EM development and EM residency program development in Jordan	Yes; rotations c/w IFEM recommendations*; no standardized courses and no research/scholarly activity requirements mentioned	1-year fellowship after completion of family medicine	Yes; UK, USA	1989
Aghababian et al.	Armenia	Development of EMS for trauma in Armenia and residency program	General description of residency program; no details on curriculum; includes course on trauma and prehospital care	n/a	Yes; USA	1995
Aksay et al.	Turkey	EM residency training in Turkey; describes the structure and content of EM programs across Turkey (change from 3 to 5 year program in 2002)	Yes; lists duration of program and extracurricular activities; research activities; curriculum generally c/w IFEM recommendations*	Initially 3, now 5 years since 2002; direct entry	n/a	1993
Binam et al.	Cameroon	EM training for MDs in Cameroon	General description of training program; no details on curriculum	2 years; for any MD with >2 years of experience	Yes; France	2001
Cevik et al.	Turkey	Overview of EM in Turkey and description of first residency program; describes the structure and development of EM programs across Turkey	Yes; lists duration of program and extracurricular activities; research activities; curriculum generally c/w IFEM recommendations*	Initially 3, now 5 years since 2002; direct entry	n/a	1993
Clem et al.	China	EM development in China and residency program	Yes; lists rotations and duration of program; curriculum generally c/w IFEM recommendations*; BLS/ACLS training	5 years; direct entry	Yes; USA	1998
Curry et al.	Papua New Guinea	EM development and training in PNG; masters in EM	No details on curriculum; mentions research/scholarly activity; ATLS/prehospital training	4 to 6 years; master’s degree	Yes; Australia	
Keyes et al.	Costa Rica	EM training in Costa Rica; describes development of residency program and challenges	No details on curriculum	3 years; direct entry	Yes	1994
Lasseter et al.	Bosnia	Overview of EM in Bosnia; describes residency program	Yes; lists duration and rotations; curriculum generally c/w IFEM recommendations*; note: general surgery rotation not listed	4 years; direct entry	n/a	1994
Partridge et al.	Cuba	Overview of EM in Cuba, update	Overview of EM and ICU training; no duration or details of training	4 years; direct entry or 3 years for specialists	n/a	2000
Rahman et al.	Malaysia	EM in Malaysia overall and EM training	Yes; lists rotations and duration; curriculum generally c/w IFEM recommendations*; BLS/ACLS/ATLS training provided	4 years; master’s degree	Yes	1998
Swanson et al.	Peru	EM training in Peru; residency program described; discusses challenges	Yes; lists and describes some rotations; mentions research/journal club activities; curriculum generally c/w IFEM recommendations*	3 years; direct entry	Yes	1993
Symmons et al.	Papua New Guinea	EM development and training in PNG; masters in EM	Discusses a medical superintendent’s observed need for EM training; briefly discusses the structure of M. Med. in EM in Papua New Guinea; research activities	4 to 6 years; master’s degree	Yes; Australia	2003
Tannebaum et al.	Brazil	Overview of EM in Brazil	Yes; lists duration and rotations; journal club activities; curriculum generally c/w IFEM recommendations*; note: general surgery rotation not listed	2 years; direct entry	n/a	1996
Wallis et al.	South Africa	Overview of EM in South Africa; describes training program and path to certification	No details on curriculum; mentions research/scholarly activity and journal club	4 years; direct entry	Yes	Late 1990s
Webb et al.	Ecuador	Overview of EM in Ecuador; short description of residency program	Lists rotations; no details on other aspects of curriculum; curriculum generally c/w IFEM recommendations*	3 years; direct entry	Yes	1995

*International federation for emergency medicine model curriculum for emergency medicine specialists^3^.

Findings are summarized below, in narrative form, under the headings: ‘General program details’, ‘Curriculum’, ‘International partnerships and exchanges’ and ‘Graduate retention’. Table 1 provides a summary of the EM training program details available from the 16 articles. Table 2 provides a summary of the Results section.

**Table 2:** Summary of results

**Theme**	**Description**
General program details	Countries described in articles meeting final inclusion criteria:
	Malaysia, Turkey, South Africa, Peru, Armenia, Cameroon, China, Costa Rica, Ecuador, Jordan, Papua New Guinea
	Number of programs per country:
	1 per country with the exception of:
	Malaysia 16, Turkey 14, South Africa 4, Peru 2
	Duration and structure:
	1-2 year fellowship programs for general practitioners, 2–5 year direct entry after medical school, 4–6 year master’s degree programs
	Please see Table 1 for details
Curriculum	Two traditional models of EM systems: Anglo-American and Franco-German (see text for details)
	Modeling of curriculum based on existing developed EM training programs:
	Bosnia, Turkey, Brazil, Costa Rica – used the North American training program curricula to inform their own program development with variable modifications to suit local needs and resources
	Cameroon – guidance from the French training program for own program development
	China, Armenia – advice from US partners but original curriculum developed to suit local needs
	No particular model described for the remaining countries
	Curriculum details and other scholarly activities:
	See Table 1 and text for further details
International partnerships/exchanges	Existing international partnerships/exchanges:
	Bosnia, Armenia, China – USA
	Jordan – UK, USA
	Cameroon – France
	Malaysia, Papua New Guinea – Australia
	Costa Rica – USA, Latin American collaborations
	Peru – USA, Colombia, other Latin American collaborations
	South Africa - UK
	None mentioned for the remaining countries.
	See Table 1 and text for further details
Graduate retention	Discussed or identified as an ongoing issue:
	Papua New Guinea – low retention; ongoing problem
	Malaysia, Costa Rica, Peru, South Africa – high retention
	No graduate retention strategies discussed for the remaining countries

### General program details

The majority of articles indicate there is only one EM program per country, with programs ranging in size from 3 to 30 trainees per year, and total graduates to date varying widely from 8 to 250. Training programs are structured in three main ways: 1-2-year fellowship programs for general physicians, 2-5-year programs with direct entry into EM residency after medical school and 4-6-year master’s degree programs.

### Curriculum

Globally, there are many variations of EM systems and EM training models, but traditionally EM training and practice have been in either of two main system models. The first is the Anglo-American system with skilled Emergency Department (ED) physicians and pre-hospital emergency medical services utilizing paramedics. The second is the Franco-German system, with a highly developed pre-hospital emergency physician service, but only a basic organization of hospital-based emergency medicine [5]. Since the roles and responsibilities of emergency physicians in these systems differ substantially because of the larger impact of the prehospital care in the Franco-German model, the training needs may differ to some degree as well. Moreover, the Franco-German model of EM systems tends to triage emergencies to particular subspecialty areas upon arrival to the hospital (orthopedics, general surgery, obstetrics/gynecology, etc.), and therefore ED physicians in such systems are not exposed to nor require training in many subspecialty emergencies. As a result, the EM training needs in this system may differ substantially from the training received in the Anglo-American EM system, which provides care for a broader scope of emergencies.

Seven countries report modeling of their curriculum after established EM systems and/or training programs. The Bosnian curriculum is modeled specifically after the Society for Academic Emergency Medicine core curriculum [6], though the article doesn’t mention whether this was modified in any way for the local context. Turkey and Brazil also chose to adapt the mature and tested North American (NA) EM training model [7]. Both the Turkish and Brazilian curricula underwent many modifications to better suit the local health-care needs [8]. Similarly, the Costa Rican curriculum was developed after studying a large number of formats from NA that were then adapted to the Costa Rican context [9].

Of the remaining three articles providing information on curriculum modeling, only the one in Cameroon is influenced by the significantly pre-hospital care-based French EM system [10]. Finally, in China and Armenia the curricula were fostered to specifically address local needs with input from US partners, but neither seemed to adopt a specific EM model [11,12]. The remaining articles do not discuss modeling after any particular curriculum or EM system.

In terms of curriculum specifics, only nine of the articles describe the components of the EM residency curriculum to some degree. The majority simply outlines the duration of training, seven list rotations (rarely with duration), and six describe extracurricular activities such as standardized advanced life support/trauma courses. Four comment on research activities and journal clubs (see Table 1).

The recently published International Federation for Emergency Medicine (IFEM) Model Curriculum for Emergency Specialists [13] is intended to provide standard, globally recognized guidelines for educational programs in emergency medicine. Depending on local resources, the implementation of these guidelines will be variable. Though the training structure or content are not explicit, the IFEM model provides a summary of the core training requirements of well-established existing training programs. It makes the following recommendations: that a program take place in an appropriate clinical setting, with adequate resources for best practice, supervision of trainees, ongoing evaluation and feedback and a list of core competencies for each year of training. The recommended overall duration of training should be at least 3 years, including training in critical care, surgery and subspecialties, internal medicine, pediatrics, prehospital/disaster medicine and emergency medicine as well as, a component of scholarly activity or research. We indicate consistency with the IFEM model where sufficient curriculum details were provided (see Table 1).

### International partnerships and exchanges

Eleven articles discussed international partnerships and exchanges as an important component of the process of developing an EM training program. The nature and extent of these partnerships varied from country to country.

In Bosnia, there was an in-country training program for emergency MDs in partnership with the International Medical Corps (IMC) Emergency Medicine Training Project at Zenica Regional Hospital. This partnership consisted of American board-certified MDs training Bosnian physicians with daily educational activities, weekly didactic sessions and clinical bedside supervision [6].

In Jordan, initial EM training courses were developed in consultation with an American emergency physician [14]. Subsequently, three Jordanian physicians and three nurses from the initial training group spent 3 months at Brooke Army Medical Center in the USA where they participated in a clinical and didactic experience in the ED and ICU. More recently, after completing the domestic 7-year program, family physicians undertaking EM specialty training have been sent to either the UK (Royal Infirmary of Edinburgh or University Hospital London) or the US (Pennsylvania State University) for a 1-year fellowship. During this time, they receive once-weekly didactic sessions as well as complete practical training modules.

Armenia developed a collaborative effort between the Boston University Medical Center, the University of Massachusetts Medical Center, the Armenian Ministry of Health and the Emergency Hospital of Yerevan, Armenia. A program director and assistant with previous EM experience and training in the USA were appointed to lead the initial efforts. The collaboration led to the development of an EM curriculum translated into Armenian and Russian and ongoing exchanges between the Armenian Emergency Hospital and partner hospitals [12].

Cameroon, which partnered with the *Mission d’Aide et de Coopération française au Cameroun,* provided trainees with a 2-month internship with the emergency medical services in France. As part of their internship, they also received first-aid training and certification as first-aid instructors [10].

In China, EM development has been ongoing with international partners providing in-country training. Three American emergency physicians and two nurse managers offered consultation and intermittent teaching and administrative support for a total of 7 months over the span of 2 years at Sir Run Run Shaw Hospital in the Zhejiang Province. Their goals included observation, identification and development of a basic framework of emergency care at this hospital [11].

Papua New Guinea has had an ongoing collaboration with Australian physicians through the Australian Agency for International Development (AusAID). AusAID initially supported an emergency physician in residence in Papua New Guinea and 14-day visits from seven other EM physicians as a limited project. Following this initial project, the University of Papua New Guinea established a Senior Lecturer position in Emergency Medicine, with several US emergency physicians rotating through since [15,16].

In Malaysia, a master’s degree in Emergency Medicine was initiated in 1986 in collaboration with the University of Sydney. In an effort to ensure that the EM training program at the University Sains Malaysia maintains international standards, they have been networking with international EM bodies and practitioners including participation of international EM professionals in the annual professional examinations as external examiners [17].

Costa Rica had two stages of collaboration with American physicians [9]. Initially, an American EM physician provided in-country training to 21 local faculty members who would subsequently train the local residents. In the second stage, a regionally appropriate curriculum was developed by a core group of Costa Rican physician educators with assistance from American emergency medicine specialists. Costa Rica has also partnered with several Latin American countries to provide exchanges to benefit from local knowledge and experience by sharing best practices as applied in their region. The authors note that the initial stage of this collaboration, which focused on local faculty preparation for the residency program, was key to the program’s ultimate success.

In Peru, residents spend 1 month in either Colombia or the US for exposure to organized trauma systems during the course of their training. The Peruvian Society of Emergency Medicine and Disasters (SPMED) was established in partnership with Colombia and the USA, and it further emphasized collaboration with international EM organizations by forming the Latin-American Association of Cooperation in Medical Emergencies and Disasters [18].

The article on South African EM training only notes that South African EM residents’ diplomas receive reciprocity with the Fellowship in Immediate Medical Care of the Royal College of Surgeons in Edinburgh; however, no details of what this entails are provided [19]. No partnerships or exchanges are mentioned in the articles on Turkey, Cuba and Brazil [7,8,20,21].

### Graduate retention

Despite the hope that developing quality EM training in-country may help to combat the loss of physicians who leave to undertake such training elsewhere, few articles provide information on graduate retention. While Costa Rica, Peru, South Africa, Turkey and Malaysia report graduate retention rates to be rather high [9,17-20], the experience in Papua New Guinea is quite different; many trainees leave to work in New Zealand or Australia where the financial reimbursement and work conditions are reported to be superior [15,16].

## Discussion

To our knowledge, this is the first review of this type and as such provides some insight into the experiences of various resource-limited health-care systems in initiating EM training programs. Common among the majority of the new programs was the adaptation of components of well-established EM program curricula, principally those of HICs and Anglo-American EM systems, as a foundation for their own curriculum development. With the continued success of these well-established EM training programs over the last few decades, these curricula may be viewed as a reliable base. However, given the unique challenges facing many resource-limited health-care systems, adaptation to ensure training is matched with local needs, priorities and resources would seem prudent if not essential to the success of a developing EM system. Several but not all articles reported on such forms of adaptation to the local context.

The second finding, common to most of the reported programs, is the development of international partnerships, with or without exchanges between participating programs. Akin to basing curriculum development on proven models, partnerships and exchanges allow participants from developing EM programs to observe and/or experience best practices modeled by mature EM programs and to benefit from clinical and didactic teaching from established EM practitioners. In the initial stages of EM development, local clinical EM expertise may be lacking. Therefore, partnerships benefit both the first cohorts of trainees as well as the pioneering local faculty who typically come from specialized clinical backgrounds such as surgery, anesthesia or internal medicine. Nonetheless, while much can be learned from partners from mature EM system, as these are predominantly from HICs they may not have much in common with LMIC health systems (even if taking into account lessons learned at the time of initial EM development in HICs). Therefore, partnerships to share experiences among similar health systems, such as the Latin-American partnerships reported above, would seem of added benefit and worthy of further exploration.

In contrast to the apparent benefits of partnerships, the advantages of exchanges appear to be less clear with some programs suggesting they may contribute to ‘brain drain’ [15]. Given that relatively few articles have reported on graduate retention, information is lacking as to under what circumstances and to what extent exchanges contribute to the loss of EM physicians after training. The burden of physician migration on already strained LMIC health systems and implications of the ‘brain drain’ phenomenon on the continuity of EM training programs in these systems necessitate efforts to monitor and share information regarding graduate retention.

Going forward, it would be useful to attempt to fill the current gaps in the literature by encouraging further knowledge and experience exchange. This could be attempted through focused surveys of newly established EM programs and/or development of a standardized format for reporting on the successes and pitfalls in curriculum development, international partnerships/exchanges as well as attempts at thwarting brain drain and encouraging graduate retention.

### Limitations

Despite the breadth of our search, relatively few articles met all of the inclusion criteria, with the majority reporting the experiences of UMICs in establishing EM programs. As noted by Arnold, “the successful development of EM relies on a reasonably mature healthcare system, with development of not only a specialty for physicians but also pre-hospital services, emergency departments well-integrated into the hospital system, as well as, specialized nursing care” [22]. The paucity of literature from LMICs may reflect the relative lack of preparedness of LMIC health systems for the development and practice of EM, particularly with respect to lack of infrastructure, facilities, equipment and supplies. While the UMICs’ experience is not directly applicable to the LMIC setting, it may still provide useful building blocks for the development of EM when appropriately adapted. Such reports also provide information helpful to LMIC governments by providing insight into priorities for investment including both training and systems components that are essential to the development and provision of emergency care. Finally, although we are aware of several newly developed EM programs in Ghana, Tanzania and Botswana, as these are not yet described in the published literature, a search of the gray literature or contacting these programs directly may allow for a broader description of EM development in LMICs in the future.

The majority of programs reported in the included articles are in their infancy (<5 years) and, with the exception of the Turkish programs, updates on development of the programs since the articles were originally published were not provided. As a result, this review is limited in its ability to determine what specific aspects might result in longer-term success or failure of LMIC EM programs. More information from the individual programs would be necessary to determine this and will hopefully become available as more programs provide updates as they continue to grow and evolve. In addition, while some articles provide detailed descriptions of their training programs, many provide only a brief general overview, which further limits the ability to draw conclusions as to what aspects of the programs reported have contributed most or might be considered essential to the successful development of EM in these settings.

## Conclusions

The results of our search indicate that EM training is in its infancy in LMICs. There are few reports of these programs’ successes and limitations, including efforts to optimize graduate retention. The paucity of published literature on EM training in LMICs likely reflects the resource constraints, with the development of the specialty of EM closely tied to that of the entire health-care system. We encourage developing EM training programs in LMICs to continue reporting on their efforts, whether successful or not, in order to help gain further understanding of the facilitators and roadblocks to such efforts. It would be helpful to gain further insight into the details of these programs including the duration, format and curriculum details including the individual rotations, number of hours, and even specific goals and objectives for each rotation. Considering the recent publication of the IFEM residency curriculum recommendations, we would encourage reporting based on these recommendations. Furthermore, we feel it is crucial to provide detailed reporting on graduate retention efforts as well as any reasons for ongoing “brain drain” in a given setting; this may identify modifiable causes for loss of trained specialists that could aid the development of preventative efforts in this area in newly developing programs. Moreover, it would be helpful to know what other educational activities and extracurricular activities are taking place within each EM training program and which of these efforts are feasible at a given stage of EM residency program development; this may include local conferences and research efforts, as well as CME opportunities. We also commend and encourage early development of collaborations and knowledge exchange with other geographically proximal programs such as the Latin-American Association of Cooperation in Medical Emergencies and Disasters in South America. These may be in the form of exchanges of trainees/fellows for short periods of time, collaborative research or regional conferences and formation of regional EM societies to help strengthen EM as a specialty in a given region. Finally, we want to underline the importance of publishing the above information in open access journals as this will make very relevant information readily available to groups entertaining the idea of developing EM training programs in low-resource settings.

## Abbreviations

EM: Emergency medicine; ED: Emergency department; ICU: Intensive care unit; LMICs: Low- and middle-income countries; HICs: High-income countries; IFEM: International federation for emergency medicine.

## Competing interests

Megan Landes is supported by a New Investigator Award from the Department of Family and Community Medicine, University of Toronto. The authors report no conflicts of interest.

## Authors’ contributions

All authors participated in the conception and design of the study. AN, ML and LPR independently reviewed the articles. LPR provided methodological oversight. AN and LPR drafted the manuscript. All authors participated in critical revisions of the manuscript, read and approved the final manuscript. AN takes responsibility for the integrity of the paper as a whole.

## Supplementary Material

Additional file 1 Database: EMBASE <1980 to 2011 Week 16>. Click here for file
