# Supplementary material for: A review of published literature on emergency medicine training programs in low- and middle-income countries
Source: Int J Emerg Med. 2013 Jul 17;6:26. doi: 10.1186/1865-1380-6-26 (PMC3718616; doi:10.1186/1865-1380-6-26)
Supplement: Additional file 1 — Database: EMBASE <1980 to 2011 Week 16>. [file 1865-1380-6-26-S1.doc]

Database: EMBASE <1980 to 2011 Week 16>

Search Strategy:

--------------------------------------------------------------------------------

1 emergency health service/ (50916)

2 emergency medicine/ (16705)

3 disaster medicine/ (335)

4 emergency medical services education/ (122)

5 emergency ward/ (33058)

6 (emergenc$ adj3 medic$).mp. (53363)

7 (disaster$ adj3 medic$).mp. (3516)

8 (emergenc$ adj3 residency).mp. (445)

9 exp medical education/ (204247)

10 exp education/ (762223)

11 exp teaching hospital/ (56934)

12 exp computer interface/ (15849)

13 exp learning/ (220958)

14 (learn$ or instruc$ or train$ or educat$ or course$ or workshop$ or work-shop$ or program$ or teach$).mp. (2246113)

15 Benin.mp. (2377)

16 (Burkina Faso or Burkina Fasso or Upper Valto).mp. (2371)

17 (Burundi or Urundi).mp. (636)

18 (Central African Republic or Ubangi-Shari or Central African Empire).mp. (786)

19 Chad.mp. (783)

20 (Comoros or Comores).mp. (243)

21 (congo or zaire).mp. (9553)

22 Eritrea.mp. (306)

23 (Ethiopia or Abyssinia or Italian East Africa or fdre).mp. (6148)

24 Gambia.mp. (2216)

25 (Ghana or Gold Coast).mp. (4838)

26 Guinea.mp. (137075)

27 (Kenya or British East Africa).mp. (11006)

28 Liberia.mp. (751)

29 (Madagascar or Malagasy Republic).mp. (2896)

30 (Malawi or British Central African Protectorate or Nyasaland).mp. (3179)

31 (Mali or French Sudan or Sudanese Republic).mp. (2285)

32 Mauritania.mp. (412)

33 (Mozambique or Portuguese East Africa).mp. (1776)

34 Niger.mp. (10069)

35 (Rwanda or Ruanda or German East Africa).mp. (1532)

36 Sierra Leone.mp. (826)

37 ((Somalia or Somali) adj1 Republic).mp. (5)

38 (Tanzania or United Republic of Tanganyika or Zanzibar).mp. (7450)

39 (Togo or Togolese Republic or togoland).mp. (1023)

40 Uganda.mp. (7295)

41 (Zambia or Northern Rhodesia).mp. (3114)

42 (Zimbabwe or Rhodesia).mp. [mp=title, abstract, subject headings, heading word, drug trade name, original title, device manufacturer, drug manufacturer] (4850)

43 (cambodia or Khmer Republic or Kampuchea).mp. (2326)

44 (Democratic People's Republic of Korea or north korea or DPRK).mp. (325)

45 (Lao PDR or Lao People's Democratic Republic or Laos).mp. (1301)

46 (Myanmar or Myanma or burma).mp. (1758)

47 (Vietnam or srv).mp. (7331)

48 (Kyrgyz Republic or Kyrgyzstan or Kirghiz Soviet Socialist Republic).mp. (1121)

49 (Tajikistan or Tajik Soviet Socialist Republic).mp. (620)

50 (Uzbekistan or Uzbek Soviet Socialist Republic).mp. (1877)

51 Haiti.mp. (1999)

52 Yemen.mp. (1173)

53 Afghanistan.mp. (3052)

54 (Bangladesh or East Bengal or East Pakistan).mp. (7792)

55 Nepal.mp. (5038)

56 Angola.mp. (816)

57 Cameroon.mp. (4072)

58 Cape Verde.mp. (291)

59 Republic of Congo.mp. (2339)

60 (Cote d'Ivoire or Ivory Coast).mp. (3057)

61 (Lesotho or Basutoland).mp. (355)

62 Nigeria.mp. (22044)

63 "Sao Tome and Principe".mp. (90)

64 Sudan.mp. (5739)

65 Swaziland.mp. (494)

66 (China or PRC).mp. (95061)

67 (Indonesia or Netherlands East Indies or Dutch East Indies).mp. (8223)

68 (Kiribati or Gilbert Islands).mp. (86)

69 (Marshall islands or rmi or "Trust Territory of the Pacific Islands").mp. (1045)

70 (Micronesia or Trust Territory of the Pacific Islands or Ponape or Truk or yap Districts or fsm).mp. (1135)

71 Mongolia.mp. (2016)

72 ((Papua New Guinea or png or Territory of Papua) and New Guinea).mp. (4696)

73 Philippines.mp. (7126)

74 Samoa.mp. (629)

75 Solomon Islands.mp. (420)

76 (Thailand or Siam).mp. (20969)

77 (Timor-Leste or East Timor or Portuguese Timor).mp. (232)

78 (Tonga or Friendly Islands).mp. (297)

79 (Vanuatu or New Hebrides).mp. (412)

80 Albania.mp. (860)

81 Armenia$.mp. (1894)

82 Azerbaijan.mp. (1348)

83 (Georgia or Georgian Soviet Socialist Republic).mp. (6637)

84 Kosovo.mp. (517)

85 (Moldova or Moldavian Soviet Socialist Republic or Moldovan Soviet Socialist Republic).mp. (676)

86 (Turkmenistan or Turkmen Soviet Socialist Republic).mp. (603)

87 (Ukraine or Ukrainian National Republic or Ukrainian State or Ukrainian Soviet Socialist Republic).mp. (14135)

88 (Belize or British Honduras).mp. (523)

89 Bolivia.mp. (2218)

90 Ecuador.mp. (2584)

91 El Salvador.mp. (919)

92 Guatemala.mp. (2523)

93 (Guyana or British Guiana).mp. (770)

94 Honduras.mp. (1125)

95 Nicaragua.mp. (1255)

96 Paraguay.mp. (903)

97 (Djibouti or Afars or Issas or French Somaliland).mp. (251)

98 (Egypt or United Arab Republic).mp. (13167)

99 (Iran or Persia).mp. (16647)

100 Iraq.mp. (4890)

101 (Jordan or Transjordan).mp. (4094)

102 Morocco.mp. (4126)

103 (Syrian Arab Republic or Syria).mp. (1024)

104 (Tunisia or Tunisian Republic).mp. (5859)

105 (West Bank or Gaza).mp. (871)

106 Bhutan.mp. (215)

107 India.mp. (89055)

108 Maldives.mp. (132)

109 Pakistan.mp. (12724)

110 (Sri Lanka or Serendib or Ceylon).mp. (4798)

111 (Africa or Asia or Caribbean or West Indies or South America or Latin America or Central America).mp. (163588)

112 developing countries/ (65335)

113 ((developing or less* developed or under developed or underdeveloped or middle income or low* income or underserved or under served or deprived or poor*) adj (countr* or nation? or population? or world)).mp. (91320)

114 ((developing or less* developed or under developed or underdeveloped or middle income or low* income) adj (economy or economies)).mp. (212)

115 (low* adj (gdp or gnp or gross domestic or gross national)).mp. (113)

116 (low adj3 middle adj3 countr*).mp. (1435)

117 (lmic or lmics or third world or lami countr*).mp. (2921)

118 transitional countr*.mp. (80)

119 or/9-14 (2452031)

120 or/1-8 (112890)

121 or/15-118 (751117)

122 119 and 120 and 121 (1907)

123 "201028".em. (24531)

124 "201029".em. (36901)

125 "20103*".em. (271359)

126 "20104*".em. (221580)

127 "20105*".em. (50497)

128 "2011*".em. (353326)

129 or/123-128 (958194)

130 122 and 129 (329)

Database: Ovid MEDLINE(R) In-Process & Other Non-Indexed Citations <April 25, 2011>

Search Strategy:

--------------------------------------------------------------------------------

1 Emergency Medicine/ (0)

2 Disaster Medicine/ (0)

3 exp Emergency Medical Services/ (0)

4 (emergenc$ adj3 medic$).mp. (1154)

5 (disaster$ adj3 medic$).mp. (42)

6 (emergenc$ adj3 residency).mp. (29)

7 or/1-6 (1186)

8 exp Education, Medical/ (0)

9 ed.fs. (38)

10 exp Education/ (3)

11 exp teaching/ (0)

12 exp teaching materials/ (1)

13 exp computer assisted instruction/ (0)

14 exp user computer interface/ (0)

15 exp learning/ (1)

16 (learn$ or instruc$ or train$ or educat$ or course$ or workshop$ or work-shop$ or program$ or teach$).mp. (67957)

17 or/8-16 (67975)

18 Benin.mp. (73)

19 (Burkina Faso or Burkina Fasso or Upper Valto).mp. [mp=protocol supplementary concept, rare disease supplementary concept, title, original title, abstract, name of substance word, subject heading word, unique identifier] (69)

20 (Burundi or Urundi).mp. [mp=protocol supplementary concept, rare disease supplementary concept, title, original title, abstract, name of substance word, subject heading word, unique identifier] (9)

21 (Central African Republic or Ubangi-Shari or Central African Empire).mp. [mp=protocol supplementary concept, rare disease supplementary concept, title, original title, abstract, name of substance word, subject heading word, unique identifier] (7)

22 Chad.mp. (22)

23 (Comoros or Comores).mp. [mp=protocol supplementary concept, rare disease supplementary concept, title, original title, abstract, name of substance word, subject heading word, unique identifier] (3)

24 (congo or Zaire).mp. [mp=protocol supplementary concept, rare disease supplementary concept, title, original title, abstract, name of substance word, subject heading word, unique identifier] (222)

25 Eritrea.mp. (11)

26 (Ethiopia or Abyssinia or Italian East Africa or fdre).mp. [mp=protocol supplementary concept, rare disease supplementary concept, title, original title, abstract, name of substance word, subject heading word, unique identifier] (227)

27 Gambia.mp. (53)

28 (Ghana or Gold Coast).mp. [mp=protocol supplementary concept, rare disease supplementary concept, title, original title, abstract, name of substance word, subject heading word, unique identifier] (270)

29 Guinea.mp. (1823)

30 (Kenya or British East Africa).mp. [mp=protocol supplementary concept, rare disease supplementary concept, title, original title, abstract, name of substance word, subject heading word, unique identifier] (390)

31 Liberia.mp. (18)

32 (Madagascar or Malagasy Republic).mp. [mp=protocol supplementary concept, rare disease supplementary concept, title, original title, abstract, name of substance word, subject heading word, unique identifier] (97)

33 (Malawi or British Central African Protectorate or Nyasaland).mp. [mp=protocol supplementary concept, rare disease supplementary concept, title, original title, abstract, name of substance word, subject heading word, unique identifier] (144)

34 (Mali or French Sudan or Sudanese Republic).mp. [mp=protocol supplementary concept, rare disease supplementary concept, title, original title, abstract, name of substance word, subject heading word, unique identifier] (80)

35 Mauritania.mp. (7)

36 (Mozambique or Portuguese East Africa).mp. [mp=protocol supplementary concept, rare disease supplementary concept, title, original title, abstract, name of substance word, subject heading word, unique identifier] (82)

37 Niger.mp. (426)

38 (Rwanda or Ruanda or German East Africa).mp. [mp=protocol supplementary concept, rare disease supplementary concept, title, original title, abstract, name of substance word, subject heading word, unique identifier] (49)

39 (Senegal or Senegambia or Mali Federation).mp. [mp=protocol supplementary concept, rare disease supplementary concept, title, original title, abstract, name of substance word, subject heading word, unique identifier] (73)

40 Sierra Leone.mp. (36)

41 ((Somalia or Somali) adj1 Republic).mp. (0)

42 (Tanzania or United Republic of Tanganyika or Zanzibar).mp. [mp=protocol supplementary concept, rare disease supplementary concept, title, original title, abstract, name of substance word, subject heading word, unique identifier] (289)

43 (Togo or Togolese Republic or togoland).mp. [mp=protocol supplementary concept, rare disease supplementary concept, title, original title, abstract, name of substance word, subject heading word, unique identifier] (18)

44 Uganda.mp. (352)

45 (Zambia or Northern Rhodesia).mp. [mp=protocol supplementary concept, rare disease supplementary concept, title, original title, abstract, name of substance word, subject heading word, unique identifier] (114)

46 (Zimbabwe or Rhodesia).mp. [mp=protocol supplementary concept, rare disease supplementary concept, title, original title, abstract, name of substance word, subject heading word, unique identifier] (104)

47 (Cambodia or Khmer Republic or Kampuchea).mp. [mp=protocol supplementary concept, rare disease supplementary concept, title, original title, abstract, name of substance word, subject heading word, unique identifier] (57)

48 (Democratic People's Republic of Korea or north korea or DPRK).mp. [mp=protocol supplementary concept, rare disease supplementary concept, title, original title, abstract, name of substance word, subject heading word, unique identifier] (14)

49 (Lao PDR or Lao People's Democratic Republic or Laos).mp. [mp=protocol supplementary concept, rare disease supplementary concept, title, original title, abstract, name of substance word, subject heading word, unique identifier] (56)

50 (Myanmar or Myanma or burma).mp. [mp=protocol supplementary concept, rare disease supplementary concept, title, original title, abstract, name of substance word, subject heading word, unique identifier] (65)

51 (Vietnam or SRV).mp. [mp=protocol supplementary concept, rare disease supplementary concept, title, original title, abstract, name of substance word, subject heading word, unique identifier] (306)

52 (Kyrgyz Republic or Kyrgyzstan or Kirghiz Soviet Socialist Republic).mp. [mp=protocol supplementary concept, rare disease supplementary concept, title, original title, abstract, name of substance word, subject heading word, unique identifier] (11)

53 (Tajikistan or Tajik Soviet Socialist Republic).mp. [mp=protocol supplementary concept, rare disease supplementary concept, title, original title, abstract, name of substance word, subject heading word, unique identifier] (13)

54 (Uzbekistan or Uzbek Soviet Socialist Republic).mp. [mp=protocol supplementary concept, rare disease supplementary concept, title, original title, abstract, name of substance word, subject heading word, unique identifier] (16)

55 Haiti.mp. (105)

56 Yemen.mp. (62)

57 Afghanistan.mp. (149)

58 (Bangladesh or East Bengal or East Pakistan).mp. [mp=protocol supplementary concept, rare disease supplementary concept, title, original title, abstract, name of substance word, subject heading word, unique identifier] (232)

59 Nepal.mp. (208)

60 Angola.mp. (28)

61 Cameroon.mp. (137)

62 Cape Verde.mp. (5)

63 Republic of Congo.mp. (36)

64 (Cote d'Ivoire or Ivory Coast).mp. [mp=protocol supplementary concept, rare disease supplementary concept, title, original title, abstract, name of substance word, subject heading word, unique identifier] (59)

65 (Lesotho or Basutoland).mp. [mp=protocol supplementary concept, rare disease supplementary concept, title, original title, abstract, name of substance word, subject heading word, unique identifier] (10)

66 Nigeria.mp. (747)

67 "Sao Tome and Principe".mp. [mp=protocol supplementary concept, rare disease supplementary concept, title, original title, abstract, name of substance word, subject heading word, unique identifier] (0)

68 Sudan.mp. (238)

69 Swaziland.mp. (25)

70 (China or PRC).mp. [mp=protocol supplementary concept, rare disease supplementary concept, title, original title, abstract, name of substance word, subject heading word, unique identifier] (3618)

71 (Indonesia or Netherlands East Indies or Dutch East Indies).mp. [mp=protocol supplementary concept, rare disease supplementary concept, title, original title, abstract, name of substance word, subject heading word, unique identifier] (215)

72 (Kiribati or Gilbert Islands).mp. [mp=protocol supplementary concept, rare disease supplementary concept, title, original title, abstract, name of substance word, subject heading word, unique identifier] (4)

73 (Marshall islands or rmi or Trust Territory of the Pacific Islands).mp. [mp=protocol supplementary concept, rare disease supplementary concept, title, original title, abstract, name of substance word, subject heading word, unique identifier] (23)

74 (Micronesia or Trust Territory of the Pacific Islands or Ponape or Truk or yap Districts or fsm).mp. [mp=protocol supplementary concept, rare disease supplementary concept, title, original title, abstract, name of substance word, subject heading word, unique identifier] (38)

75 Mongolia.mp. (93)

76 ((Papua New Guinea or png or Territory of Papua) and New Guinea).mp. [mp=protocol supplementary concept, rare disease supplementary concept, title, original title, abstract, name of substance word, subject heading word, unique identifier] (55)

77 Philippines.mp. (157)

78 Samoa.mp. (25)

79 Solomon Islands.mp. (22)

80 (Thailand or Siam).mp. [mp=protocol supplementary concept, rare disease supplementary concept, title, original title, abstract, name of substance word, subject heading word, unique identifier] (503)

81 (Timor-Leste or East Timor or Portuguese Timor).mp. [mp=protocol supplementary concept, rare disease supplementary concept, title, original title, abstract, name of substance word, subject heading word, unique identifier] (5)

82 (Tonga or Friendly Islands).mp. [mp=protocol supplementary concept, rare disease supplementary concept, title, original title, abstract, name of substance word, subject heading word, unique identifier] (25)

83 (Vanuatu or New Hebrides).mp. [mp=protocol supplementary concept, rare disease supplementary concept, title, original title, abstract, name of substance word, subject heading word, unique identifier] (13)

84 Albania.mp. (29)

85 Armenia$.mp. (58)

86 Azerbaijan.mp. (25)

87 (Georgia or Georgian Soviet Socialist Republic).mp. [mp=protocol supplementary concept, rare disease supplementary concept, title, original title, abstract, name of substance word, subject heading word, unique identifier] (272)

88 Kosovo.mp. (21)

89 (Moldova or Moldavian Soviet Socialist Republic or Moldovan Soviet Socialist Republic).mp. [mp=protocol supplementary concept, rare disease supplementary concept, title, original title, abstract, name of substance word, subject heading word, unique identifier] (4)

90 (Turkmenistan or Turkmen Soviet Socialist Republic).mp. [mp=protocol supplementary concept, rare disease supplementary concept, title, original title, abstract, name of substance word, subject heading word, unique identifier] (4)

91 (Ukraine or Ukrainian National Republic or Ukrainian State or Ukrainian Soviet Socialist Republic).mp. [mp=protocol supplementary concept, rare disease supplementary concept, title, original title, abstract, name of substance word, subject heading word, unique identifier] (56)

92 (Belize or British Honduras).mp. [mp=protocol supplementary concept, rare disease supplementary concept, title, original title, abstract, name of substance word, subject heading word, unique identifier] (25)

93 Bolivia.mp. (56)

94 Ecuador.mp. (83)

95 El Salvador.mp. (18)

96 Guatemala.mp. (94)

97 (Guyana or British Guiana).mp. [mp=protocol supplementary concept, rare disease supplementary concept, title, original title, abstract, name of substance word, subject heading word, unique identifier] (33)

98 Honduras.mp. (40)

99 Nicaragua.mp. (49)

100 Paraguay.mp. (22)

101 (Djibouti or Afars or Issas or French Somaliland).mp. [mp=protocol supplementary concept, rare disease supplementary concept, title, original title, abstract, name of substance word, subject heading word, unique identifier] (4)

102 (Egypt or United Arab Republic).mp. [mp=protocol supplementary concept, rare disease supplementary concept, title, original title, abstract, name of substance word, subject heading word, unique identifier] (454)

103 (Iran or Persia).mp. [mp=protocol supplementary concept, rare disease supplementary concept, title, original title, abstract, name of substance word, subject heading word, unique identifier] (677)

104 Iraq.mp. (214)

105 (Jordan or Transjordan).mp. [mp=protocol supplementary concept, rare disease supplementary concept, title, original title, abstract, name of substance word, subject heading word, unique identifier] (249)

106 Morocco.mp. (104)

107 (Syrian Arab Republic or Syria).mp. [mp=protocol supplementary concept, rare disease supplementary concept, title, original title, abstract, name of substance word, subject heading word, unique identifier] (48)

108 (Tunisia or Tunisian Republic).mp. [mp=protocol supplementary concept, rare disease supplementary concept, title, original title, abstract, name of substance word, subject heading word, unique identifier] (123)

109 (West Bank or Gaza).mp. [mp=protocol supplementary concept, rare disease supplementary concept, title, original title, abstract, name of substance word, subject heading word, unique identifier] (36)

110 Bhutan.mp. (15)

111 India.mp. (2658)

112 Maldives.mp. (2)

113 Pakistan.mp. (487)

114 (Sri Lanka or Serendib or Ceylon).mp. [mp=protocol supplementary concept, rare disease supplementary concept, title, original title, abstract, name of substance word, subject heading word, unique identifier] (152)

115 (Africa or Asia or Caribbean or West Indies or South America or Latin America or Central America).mp. (5060)

116 developing countries/ (2)

117 ((developing or less* developed or under developed or underdeveloped or middle income or low* income or underserved or under served or deprived or poor*) adj (countr* or nation? or population? or world)).mp. (2489)

118 ((developing or less* developed or under developed or underdeveloped or middle income or low* income) adj (economy or economies)).mp. (18)

119 (low* adj (gdp or gnp or gross domestic or gross national)).mp. (3)

120 (low adj3 middle adj3 countr*).mp. (206)

121 (lmic or lmics or third world or lami countr*).mp. (131)

122 transitional countr*.mp. (9)

123 or/18-122 (22103)

124 7 and 17 and 123 (36)

125 "201006*".ed. (108885)

126 "201007*".ed. (100614)

127 "201008*".ed. (2640)

128 "201009*".ed. (3354)

129 "201010*".ed. (1915)

130 "201011*".ed. (2169)

131 "201012*".ed. (2650)

132 2011*.ed. (95553)

133 or/125-132 (317780)

134 124 and 133 (10)

Database: Ovid MEDLINE(R) <1948 to April Week 2 2011>

Search Strategy:

--------------------------------------------------------------------------------

1 Emergency Medicine/ (8325)

2 Disaster Medicine/ (260)

3 exp Emergency Medical Services/ (77431)

4 (emergenc$ adj3 medic$).mp. (45693)

5 (disaster$ adj3 medic$).mp. (990)

6 (emergenc$ adj3 residency).mp. (403)

7 or/1-6 (91673)

8 exp Education, Medical/ (114136)

9 ed.fs. (189321)

10 exp Education/ (536418)

11 exp teaching/ (58121)

12 exp teaching materials/ (85339)

13 exp computer assisted instruction/ (7790)

14 exp user computer interface/ (21399)

15 exp learning/ (241628)

16 (learn$ or instruc$ or train$ or educat$ or course$ or workshop$ or work-shop$ or program$ or teach$).mp. (1647344)

17 or/8-16 (1949799)

18 Benin.mp. (1945)

19 (Burkina Faso or Burkina Fasso or Upper Valto).mp. [mp=protocol supplementary concept, rare disease supplementary concept, title, original title, abstract, name of substance word, subject heading word, unique identifier] (2057)

20 (Burundi or Urundi).mp. [mp=protocol supplementary concept, rare disease supplementary concept, title, original title, abstract, name of substance word, subject heading word, unique identifier] (610)

21 (Central African Republic or Ubangi-Shari or Central African Empire).mp. [mp=protocol supplementary concept, rare disease supplementary concept, title, original title, abstract, name of substance word, subject heading word, unique identifier] (749)

22 Chad.mp. (711)

23 (Comoros or Comores).mp. [mp=protocol supplementary concept, rare disease supplementary concept, title, original title, abstract, name of substance word, subject heading word, unique identifier] (234)

24 (congo or Zaire).mp. [mp=protocol supplementary concept, rare disease supplementary concept, title, original title, abstract, name of substance word, subject heading word, unique identifier] (8585)

25 Eritrea.mp. (270)

26 (Ethiopia or Abyssinia or Italian East Africa or fdre).mp. [mp=protocol supplementary concept, rare disease supplementary concept, title, original title, abstract, name of substance word, subject heading word, unique identifier] (6024)

27 Gambia.mp. (2234)

28 (Ghana or Gold Coast).mp. [mp=protocol supplementary concept, rare disease supplementary concept, title, original title, abstract, name of substance word, subject heading word, unique identifier] (4404)

29 Guinea.mp. (143719)

30 (Kenya or British East Africa).mp. [mp=protocol supplementary concept, rare disease supplementary concept, title, original title, abstract, name of substance word, subject heading word, unique identifier] (10708)

31 Liberia.mp. (801)

32 (Madagascar or Malagasy Republic).mp. [mp=protocol supplementary concept, rare disease supplementary concept, title, original title, abstract, name of substance word, subject heading word, unique identifier] (2651)

33 (Malawi or British Central African Protectorate or Nyasaland).mp. [mp=protocol supplementary concept, rare disease supplementary concept, title, original title, abstract, name of substance word, subject heading word, unique identifier] (2922)

34 (Mali or French Sudan or Sudanese Republic).mp. [mp=protocol supplementary concept, rare disease supplementary concept, title, original title, abstract, name of substance word, subject heading word, unique identifier] (1981)

35 Mauritania.mp. (379)

36 (Mozambique or Portuguese East Africa).mp. [mp=protocol supplementary concept, rare disease supplementary concept, title, original title, abstract, name of substance word, subject heading word, unique identifier] (1562)

37 Niger.mp. (7344)

38 (Rwanda or Ruanda or German East Africa).mp. [mp=protocol supplementary concept, rare disease supplementary concept, title, original title, abstract, name of substance word, subject heading word, unique identifier] (1455)

39 (Senegal or Senegambia or Mali Federation).mp. [mp=protocol supplementary concept, rare disease supplementary concept, title, original title, abstract, name of substance word, subject heading word, unique identifier] (4919)

40 Sierra Leone.mp. (812)

41 ((Somalia or Somali) adj1 Republic).mp. (5)

42 (Tanzania or United Republic of Tanganyika or Zanzibar).mp. [mp=protocol supplementary concept, rare disease supplementary concept, title, original title, abstract, name of substance word, subject heading word, unique identifier] (6980)

43 (Togo or Togolese Republic or togoland).mp. [mp=protocol supplementary concept, rare disease supplementary concept, title, original title, abstract, name of substance word, subject heading word, unique identifier] (902)

44 Uganda.mp. (6869)

45 (Zambia or Northern Rhodesia).mp. [mp=protocol supplementary concept, rare disease supplementary concept, title, original title, abstract, name of substance word, subject heading word, unique identifier] (3090)

46 (Zimbabwe or Rhodesia).mp. [mp=protocol supplementary concept, rare disease supplementary concept, title, original title, abstract, name of substance word, subject heading word, unique identifier] (4923)

47 (Cambodia or Khmer Republic or Kampuchea).mp. [mp=protocol supplementary concept, rare disease supplementary concept, title, original title, abstract, name of substance word, subject heading word, unique identifier] (2068)

48 (Democratic People's Republic of Korea or north korea or DPRK).mp. [mp=protocol supplementary concept, rare disease supplementary concept, title, original title, abstract, name of substance word, subject heading word, unique identifier] (153)

49 (Lao PDR or Lao People's Democratic Republic or Laos).mp. [mp=protocol supplementary concept, rare disease supplementary concept, title, original title, abstract, name of substance word, subject heading word, unique identifier] (1347)

50 (Myanmar or Myanma or burma).mp. [mp=protocol supplementary concept, rare disease supplementary concept, title, original title, abstract, name of substance word, subject heading word, unique identifier] (1661)

51 (Vietnam or SRV).mp. [mp=protocol supplementary concept, rare disease supplementary concept, title, original title, abstract, name of substance word, subject heading word, unique identifier] (9746)

52 (Kyrgyz Republic or Kyrgyzstan or Kirghiz Soviet Socialist Republic).mp. [mp=protocol supplementary concept, rare disease supplementary concept, title, original title, abstract, name of substance word, subject heading word, unique identifier] (1118)

53 (Tajikistan or Tajik Soviet Socialist Republic).mp. [mp=protocol supplementary concept, rare disease supplementary concept, title, original title, abstract, name of substance word, subject heading word, unique identifier] (604)

54 (Uzbekistan or Uzbek Soviet Socialist Republic).mp. [mp=protocol supplementary concept, rare disease supplementary concept, title, original title, abstract, name of substance word, subject heading word, unique identifier] (1811)

55 Haiti.mp. (2028)

56 Yemen.mp. (1099)

57 Afghanistan.mp. (2555)

58 (Bangladesh or East Bengal or East Pakistan).mp. [mp=protocol supplementary concept, rare disease supplementary concept, title, original title, abstract, name of substance word, subject heading word, unique identifier] (6875)

59 Nepal.mp. (4454)

60 Angola.mp. (749)

61 Cameroon.mp. (3575)

62 Cape Verde.mp. (258)

63 Republic of Congo.mp. (575)

64 (Cote d'Ivoire or Ivory Coast).mp. [mp=protocol supplementary concept, rare disease supplementary concept, title, original title, abstract, name of substance word, subject heading word, unique identifier] (2752)

65 (Lesotho or Basutoland).mp. [mp=protocol supplementary concept, rare disease supplementary concept, title, original title, abstract, name of substance word, subject heading word, unique identifier] (361)

66 Nigeria.mp. (19534)

67 "Sao Tome and Principe".mp. [mp=protocol supplementary concept, rare disease supplementary concept, title, original title, abstract, name of substance word, subject heading word, unique identifier] (68)

68 Sudan.mp. (5407)

69 Swaziland.mp. (392)

70 (China or PRC).mp. [mp=protocol supplementary concept, rare disease supplementary concept, title, original title, abstract, name of substance word, subject heading word, unique identifier] (72157)

71 (Indonesia or Netherlands East Indies or Dutch East Indies).mp. [mp=protocol supplementary concept, rare disease supplementary concept, title, original title, abstract, name of substance word, subject heading word, unique identifier] (7628)

72 (Kiribati or Gilbert Islands).mp. [mp=protocol supplementary concept, rare disease supplementary concept, title, original title, abstract, name of substance word, subject heading word, unique identifier] (84)

73 (Marshall islands or rmi or Trust Territory of the Pacific Islands).mp. [mp=protocol supplementary concept, rare disease supplementary concept, title, original title, abstract, name of substance word, subject heading word, unique identifier] (812)

74 (Micronesia or Trust Territory of the Pacific Islands or Ponape or Truk or yap Districts or fsm).mp. [mp=protocol supplementary concept, rare disease supplementary concept, title, original title, abstract, name of substance word, subject heading word, unique identifier] (1186)

75 Mongolia.mp. (1719)

76 ((Papua New Guinea or png or Territory of Papua) and New Guinea).mp. [mp=protocol supplementary concept, rare disease supplementary concept, title, original title, abstract, name of substance word, subject heading word, unique identifier] (3612)

77 Philippines.mp. (6981)

78 Samoa.mp. (648)

79 Solomon Islands.mp. (362)

80 (Thailand or Siam).mp. [mp=protocol supplementary concept, rare disease supplementary concept, title, original title, abstract, name of substance word, subject heading word, unique identifier] (19712)

81 (Timor-Leste or East Timor or Portuguese Timor).mp. [mp=protocol supplementary concept, rare disease supplementary concept, title, original title, abstract, name of substance word, subject heading word, unique identifier] (175)

82 (Tonga or Friendly Islands).mp. [mp=protocol supplementary concept, rare disease supplementary concept, title, original title, abstract, name of substance word, subject heading word, unique identifier] (267)

83 (Vanuatu or New Hebrides).mp. [mp=protocol supplementary concept, rare disease supplementary concept, title, original title, abstract, name of substance word, subject heading word, unique identifier] (369)

84 Albania.mp. (667)

85 Armenia$.mp. (1639)

86 Azerbaijan.mp. (1118)

87 (Georgia or Georgian Soviet Socialist Republic).mp. [mp=protocol supplementary concept, rare disease supplementary concept, title, original title, abstract, name of substance word, subject heading word, unique identifier] (11422)

88 Kosovo.mp. (426)

89 (Moldova or Moldavian Soviet Socialist Republic or Moldovan Soviet Socialist Republic).mp. [mp=protocol supplementary concept, rare disease supplementary concept, title, original title, abstract, name of substance word, subject heading word, unique identifier] (685)

90 (Turkmenistan or Turkmen Soviet Socialist Republic).mp. [mp=protocol supplementary concept, rare disease supplementary concept, title, original title, abstract, name of substance word, subject heading word, unique identifier] (602)

91 (Ukraine or Ukrainian National Republic or Ukrainian State or Ukrainian Soviet Socialist Republic).mp. [mp=protocol supplementary concept, rare disease supplementary concept, title, original title, abstract, name of substance word, subject heading word, unique identifier] (14527)

92 (Belize or British Honduras).mp. [mp=protocol supplementary concept, rare disease supplementary concept, title, original title, abstract, name of substance word, subject heading word, unique identifier] (506)

93 Bolivia.mp. (2080)

94 Ecuador.mp. (2436)

95 El Salvador.mp. (861)

96 Guatemala.mp. (2517)

97 (Guyana or British Guiana).mp. [mp=protocol supplementary concept, rare disease supplementary concept, title, original title, abstract, name of substance word, subject heading word, unique identifier] (739)

98 Honduras.mp. (1083)

99 Nicaragua.mp. (1157)

100 Paraguay.mp. (795)

101 (Djibouti or Afars or Issas or French Somaliland).mp. [mp=protocol supplementary concept, rare disease supplementary concept, title, original title, abstract, name of substance word, subject heading word, unique identifier] (243)

102 (Egypt or United Arab Republic).mp. [mp=protocol supplementary concept, rare disease supplementary concept, title, original title, abstract, name of substance word, subject heading word, unique identifier] (11053)

103 (Iran or Persia).mp. [mp=protocol supplementary concept, rare disease supplementary concept, title, original title, abstract, name of substance word, subject heading word, unique identifier] (10571)

104 Iraq.mp. (4616)

105 (Jordan or Transjordan).mp. [mp=protocol supplementary concept, rare disease supplementary concept, title, original title, abstract, name of substance word, subject heading word, unique identifier] (3042)

106 Morocco.mp. (3619)

107 (Syrian Arab Republic or Syria).mp. [mp=protocol supplementary concept, rare disease supplementary concept, title, original title, abstract, name of substance word, subject heading word, unique identifier] (998)

108 (Tunisia or Tunisian Republic).mp. [mp=protocol supplementary concept, rare disease supplementary concept, title, original title, abstract, name of substance word, subject heading word, unique identifier] (5053)

109 (West Bank or Gaza).mp. [mp=protocol supplementary concept, rare disease supplementary concept, title, original title, abstract, name of substance word, subject heading word, unique identifier] (784)

110 Bhutan.mp. (206)

111 India.mp. (72069)

112 Maldives.mp. (109)

113 Pakistan.mp. (9685)

114 (Sri Lanka or Serendib or Ceylon).mp. [mp=protocol supplementary concept, rare disease supplementary concept, title, original title, abstract, name of substance word, subject heading word, unique identifier] (4484)

115 (Africa or Asia or Caribbean or West Indies or South America or Latin America or Central America).mp. (134206)

116 developing countries/ (55469)

117 ((developing or less* developed or under developed or underdeveloped or middle income or low* income or underserved or under served or deprived or poor*) adj (countr* or nation? or population? or world)).mp. (78904)

118 ((developing or less* developed or under developed or underdeveloped or middle income or low* income) adj (economy or economies)).mp. (169)

119 (low* adj (gdp or gnp or gross domestic or gross national)).mp. (98)

120 (low adj3 middle adj3 countr*).mp. (1086)

121 (lmic or lmics or third world or lami countr*).mp. (2591)

122 transitional countr*.mp. (60)

123 or/18-122 (667639)

124 7 and 17 and 123 (1306)

125 "201006*".ed. (68965)

126 "201007*".ed. (57849)

127 "201008*".ed. (59937)

128 "201009*".ed. (73398)

129 "201010*".ed. (66414)

130 "201011*".ed. (59267)

131 "201012*".ed. (58524)

132 2011*.ed. (223451)

133 or/125-132 (667805)

134 124 and 133 (122)

Database: EBM Reviews - Database of Abstracts of Reviews of Effects <2nd Quarter 2011>

Search Strategy:

--------------------------------------------------------------------------------

1 (emergenc$ adj3 medic$).mp. (109)

2 (disaster$ adj3 medic$).mp. (1)

3 (emergenc$ adj3 residency).mp. (0)

4 (learn$ or instruc$ or train$ or educat$ or course$ or workshop$ or work-shop$ or program$ or teach$).mp. (3527)

5 Benin.mp. (1)

6 (Burkina Faso or Burkina Fasso or Upper Valto).mp. (1)

7 (Burundi or Urundi).mp. (1)

8 (Central African Republic or Ubangi-Shari or Central African Empire).mp. (0)

9 Chad.mp. (0)

10 (Comoros or Comores).mp. (0)

11 (congo or Zaire).mp. (4)

12 Eritrea.mp. (0)

13 (Ethiopia or Abyssinia or Italian East Africa or fdre).mp. (5)

14 Gambia.mp. (4)

15 (Ghana or Gold Coast).mp. (4)

16 Guinea.mp. (7)

17 (Kenya or British East Africa).mp. (8)

18 Liberia.mp. (0)

19 (Madagascar or Malagasy Republic).mp. (0)

20 (Malawi or British Central African Protectorate or Nyasaland).mp. (6)

21 (Mali or French Sudan or Sudanese Republic).mp. (1)

22 Mauritania.mp. (0)

23 (Mozambique or Portuguese East Africa).mp. (3)

24 Niger.mp. (1)

25 (Rwanda or Ruanda or German East Africa).mp. (0)

26 (Senegal or Senegambia or Mali Federation).mp. (5)

27 Sierra Leone.mp. (1)

28 ((Somalia or Somali) adj1 Republic).mp. (0)

29 (Tanzania or United Republic of Tanganyika or Zanzibar).mp. (2)

30 (Togo or Togolese Republic or togoland).mp. (0)

31 Uganda.mp. (6)

32 (Zambia or Northern Rhodesia).mp. (2)

33 (Zimbabwe or Rhodesia).mp. (6)

34 (Cambodia or Khmer Republic or Kampuchea).mp. (0)

35 (Democratic People's Republic of Korea or north korea or DPRK).mp. (0)

36 (Lao PDR or Lao People's Democratic Republic or Laos).mp. (1)

37 (Myanmar or Myanma or burma).mp. (1)

38 (Vietnam or SRV).mp. (6)

39 (Kyrgyz Republic or Kyrgyzstan or Kirghiz Soviet Socialist Republic).mp. (0)

40 (Tajikistan or Tajik Soviet Socialist Republic).mp. (0)

41 (Uzbekistan or Uzbek Soviet Socialist Republic).mp. (2)

42 Haiti.mp. (1)

43 Yemen.mp. (0)

44 Yemen.mp. (0)

45 Afghanistan.mp. (2)

46 (Bangladesh or East Bengal or East Pakistan).mp. (9)

47 Nepal.mp. (2)

48 Angola.mp. (0)

49 Cameroon.mp. (1)

50 Republic of Congo.mp. (2)

51 (Cote d'Ivoire or Ivory Coast).mp. (2)

52 (Lesotho or Basutoland).mp. (0)

53 Nigeria.mp. (5)

54 "Sao Tome and Principe".mp. (1)

55 Sudan.mp. (1)

56 Swaziland.mp. (0)

57 (China or PRC).mp. (217)

58 (Indonesia or Netherlands East Indies or Dutch East Indies).mp. (4)

59 (Kiribati or Gilbert Islands).mp. (0)

60 (Marshall islands or rmi or Trust Territory of the Pacific Islands).mp. (0)

61 (Micronesia or Trust Territory of the Pacific Islands or Ponape or Truk or yap Districts or fsm).mp. (0)

62 Mongolia.mp. (0)

63 ((Papua New Guinea or png or Territory of Papua) and New Guinea).mp. (2)

64 Philippines.mp. (5)

65 Samoa.mp. (0)

66 Solomon Islands.mp. (0)

67 (Thailand or Siam).mp. (28)

68 (Timor-Leste or East Timor or Portuguese Timor).mp. (0)

69 (Tonga or Friendly Islands).mp. (0)

70 (Vanuatu or New Hebrides).mp. (0)

71 Albania.mp. (0)

72 Armenia$.mp. (0)

73 Azerbaijan.mp. (0)

74 (Georgia or Georgian Soviet Socialist Republic).mp. (3)

75 Kosovo.mp. (1)

76 (Moldova or Moldavian Soviet Socialist Republic or Moldovan Soviet Socialist Republic).mp. (0)

77 (Turkmenistan or Turkmen Soviet Socialist Republic).mp. (0)

78 (Ukraine or Ukrainian National Republic or Ukrainian State or Ukrainian Soviet Socialist Republic).mp. (2)

79 (Belize or British Honduras).mp. (0)

80 Bolivia.mp. (1)

81 Ecuador.mp. (4)

82 El Salvador.mp. (0)

83 Guatemala.mp. (2)

84 (Guyana or British Guiana).mp. (0)

85 Honduras.mp. (3)

86 Nicaragua.mp. (2)

87 Paraguay.mp. (0)

88 (Djibouti or Afars or Issas or French Somaliland).mp. (0)

89 (Egypt or United Arab Republic).mp. (12)

90 (Iran or Persia).mp. (13)

91 Iraq.mp. (3)

92 (Jordan or Transjordan).mp. (3)

93 Morocco.mp. (0)

94 (Syrian Arab Republic or Syria).mp. (0)

95 (Tunisia or Tunisian Republic).mp. (0)

96 (West Bank or Gaza).mp. (0)

97 Bhutan.mp. (0)

98 India.mp. (62)

99 Maldives.mp. (0)

100 Pakistan.mp. (11)

101 (Sri Lanka or Serendib or Ceylon).mp. (3)

102 (Africa or Asia or Caribbean or West Indies or South America or Latin America or Central America).mp. (198)

103 ((developing or less* developed or under developed or underdeveloped or middle income or low* income or underserved or under served or deprived or poor*) adj (countr* or nation? or population? or world)).mp. (187)

104 ((developing or less* developed or under developed or underdeveloped or middle income or low* income) adj (economy or economies)).mp. (0)

105 (low* adj (gdp or gnp or gross domestic or gross national)).mp. (0)

106 (low adj3 middle adj3 countr*).mp. (14)

107 (lmic or lmics or third world or lami countr*).mp. (1)

108 transitional countr*.mp. (0)

109 or/1-3 (109)

110 or/5-108 (622)

111 4 and 109 and 110 (3)

112 from 111 keep 3 (1)

Database: Ovid Healthstar <1966 to March 2011>

Search Strategy:

--------------------------------------------------------------------------------

1 Emergency Medicine/ (8493)

2 Disaster Medicine/ (249)

3 exp Emergency Medical Services/ (76457)

4 (emergenc$ adj3 medic$).mp. (44872)

5 (disaster$ adj3 medic$).mp. (1039)

6 (emergenc$ adj3 residency).mp. (405)

7 exp Education, Medical/ (108708)

8 ed.fs. (191739)

9 exp Education/ (487111)

10 exp teaching/ (53223)

11 exp teaching materials/ (74910)

12 exp computer assisted instruction/ (7702)

13 exp user computer interface/ (21065)

14 exp learning/ (120941)

15 (learn$ or instruc$ or train$ or educat$ or course$ or workshop$ or work-shop$ or program$ or teach$).mp. (1240433)

16 Benin.mp. (1326)

17 (Burkina Faso or Burkina Fasso or Upper Valto).mp. (1601)

18 (Burundi or Urundi).mp. (456)

19 (Central African Republic or Ubangi-Shari or Central African Empire).mp. (488)

20 Chad.mp. (453)

21 (Comoros or Comores).mp. (172)

22 (congo or Zaire).mp. (4742)

23 Eritrea.mp. (201)

24 (Ethiopia or Abyssinia or Italian East Africa or fdre).mp. (4542)

25 Gambia.mp. (1728)

26 (Ghana or Gold Coast).mp. (3582)

27 Guinea.mp. (16717)

28 (Kenya or British East Africa).mp. (7569)

29 Liberia.mp. (525)

30 (Madagascar or Malagasy Republic).mp. (1132)

31 (Malawi or British Central African Protectorate or Nyasaland).mp. (2489)

32 (Mali or French Sudan or Sudanese Republic).mp. (1411)

33 Mauritania.mp. (245)

34 (Mozambique or Portuguese East Africa).mp. (1127)

35 Niger.mp. (3135)

36 (Rwanda or Ruanda or German East Africa).mp. (1195)

37 (Senegal or Senegambia or Mali Federation).mp. (3238)

38 Sierra Leone.mp. (625)

39 ((Somalia or Somali) adj1 Republic).mp. (2)

40 (Tanzania or United Republic of Tanganyika or Zanzibar).mp. (5510)

41 (Togo or Togolese Republic or togoland).mp. (641)

42 Uganda.mp. (5466)

43 (Zambia or Northern Rhodesia).mp. (2393)

44 (Zimbabwe or Rhodesia).mp. (3616)

45 (Cambodia or Khmer Republic or Kampuchea).mp. (1806)

46 (Democratic People's Republic of Korea or north korea or DPRK).mp. (129)

47 (Lao PDR or Lao People's Democratic Republic or Laos).mp. (1120)

48 (Myanmar or Myanma or burma).mp. (1098)

49 (Vietnam or SRV).mp. (8072)

50 (Kyrgyz Republic or Kyrgyzstan or Kirghiz Soviet Socialist Republic).mp. (890)

51 (Tajikistan or Tajik Soviet Socialist Republic).mp. (456)

52 (Uzbekistan or Uzbek Soviet Socialist Republic).mp. (1332)

53 Haiti.mp. (1848)

54 Yemen.mp. (897)

55 Yemen.mp. (897)

56 Afghanistan.mp. (2437)

57 (Bangladesh or East Bengal or East Pakistan).mp. (5970)

58 Nepal.mp. (3714)

59 Angola.mp. (528)

60 Cameroon.mp. (2423)

61 Republic of Congo.mp. (472)

62 (Cote d'Ivoire or Ivory Coast).mp. (1936)

63 (Lesotho or Basutoland).mp. (303)

64 Nigeria.mp. (15287)

65 "Sao Tome and Principe".mp. (44)

66 Sudan.mp. (3051)

67 Swaziland.mp. (332)

68 (China or PRC).mp. (53624)

69 (Indonesia or Netherlands East Indies or Dutch East Indies).mp. (5290)

70 (Kiribati or Gilbert Islands).mp. (69)

71 (Marshall islands or rmi or Trust Territory of the Pacific Islands).mp. (502)

72 (Micronesia or Trust Territory of the Pacific Islands or Ponape or Truk or yap Districts or fsm).mp. (944)

73 Mongolia.mp. (1085)

74 ((Papua New Guinea or png or Territory of Papua) and New Guinea).mp. (2531)

75 Philippines.mp. (5337)

76 Samoa.mp. (563)

77 Solomon Islands.mp. (225)

78 (Thailand or Siam).mp. (14185)

79 (Timor-Leste or East Timor or Portuguese Timor).mp. (176)

80 (Tonga or Friendly Islands).mp. (236)

81 (Vanuatu or New Hebrides).mp. (242)

82 Albania.mp. (593)

83 Armenia$.mp. (1206)

84 Azerbaijan.mp. (865)

85 (Georgia or Georgian Soviet Socialist Republic).mp. (9847)

86 Kosovo.mp. (410)

87 (Moldova or Moldavian Soviet Socialist Republic or Moldovan Soviet Socialist Republic).mp. (554)

88 (Turkmenistan or Turkmen Soviet Socialist Republic).mp. (430)

89 (Ukraine or Ukrainian National Republic or Ukrainian State or Ukrainian Soviet Socialist Republic).mp. (12318)

90 (Belize or British Honduras).mp. (261)

91 Bolivia.mp. (1486)

92 Ecuador.mp. (1638)

93 El Salvador.mp. (675)

94 Guatemala.mp. (1973)

95 (Guyana or British Guiana).mp. (484)

96 Honduras.mp. (812)

97 Nicaragua.mp. (952)

98 Paraguay.mp. (497)

99 (Djibouti or Afars or Issas or French Somaliland).mp. (184)

100 (Egypt or United Arab Republic).mp. (7035)

101 (Iran or Persia).mp. (8062)

102 Iraq.mp. (3960)

103 (Jordan or Transjordan).mp. (2466)

104 Morocco.mp. (2367)

105 (Syrian Arab Republic or Syria).mp. (705)

106 (Tunisia or Tunisian Republic).mp. (3521)

107 (West Bank or Gaza).mp. (643)

108 Bhutan.mp. (140)

109 India.mp. (57261)

110 Maldives.mp. (64)

111 Pakistan.mp. (9349)

112 (Sri Lanka or Serendib or Ceylon).mp. (3348)

113 (Africa or Asia or Caribbean or West Indies or South America or Latin America or Central America).mp. (108281)

114 ((developing or less* developed or under developed or underdeveloped or middle income or low* income or underserved or under served or deprived or poor*) adj (countr* or nation? or population? or world)).mp. (78372)

115 ((developing or less* developed or under developed or underdeveloped or middle income or low* income) adj (economy or economies)).mp. (147)

116 (low* adj (gdp or gnp or gross domestic or gross national)).mp. (62)

117 (low adj3 middle adj3 countr*).mp. (1163)

118 (lmic or lmics or third world or lami countr*).mp. (2203)

119 transitional countr*.mp. (67)

120 developing countries/ (58026)

121 or/1-6 (89531)

122 or/16-120 (427586)

123 or/7-15 (1484411)

124 121 and 122 and 123 (1394)

125 limit 124 to index medicus (1181)

126 124 not 125 (213)

127 "201006*".ed. (43593)

128 "201007*".ed. (35887)

129 "201008*".ed. (38033)

130 "201009*".ed. (46344)

131 "201010*".ed. (33885)

132 "201011*".ed. (22845)

133 "201012*".ed. (9271)

134 2011*.ed. (118890)

135 or/127-134 (348748)

136 126 and 135 (19)

Web of Science: For these results I saved into an ENDNOTE database. I just manually saved the most recent results that were not in the last set. There did not appear to be a way to limit to certain weeks like in the other databases.

| Top of Form  # 29  Bottom of Form | | [500](http://apps.isiknowledge.com.myaccess.library.utoronto.ca/summary.do?product=WOS&doc=1&qid=58&SID=4BCl9iO2E7G18kLgIIL&search_mode=CombineSearches) | | #28 OR #27 OR #26 OR #25 OR #24 OR #23 OR #22 OR #21 OR #20 | |  |  |
| --- | --- | --- | --- | --- | --- | --- | --- |
|  |  |  |  | Databases=SCI-EXPANDED, SSCI, A&HCI, CPCI-S, CPCI-SSH Timespan=All Years | |  |  |
| |  | | --- | | | | | | | | |
| # 28 | | [231](http://apps.isiknowledge.com.myaccess.library.utoronto.ca/summary.do?product=WOS&doc=1&qid=57&SID=4BCl9iO2E7G18kLgIIL&search_mode=CombineSearches) | | #19 AND #2 AND #1 | |  |  |
|  |  |  |  | Databases=SCI-EXPANDED, SSCI, A&HCI, CPCI-S, CPCI-SSH Timespan=All Years | |  |  |
| |  | | --- | | | | | | | | |
| # 27 | | [98](http://apps.isiknowledge.com.myaccess.library.utoronto.ca/summary.do?product=WOS&doc=1&qid=56&SID=4BCl9iO2E7G18kLgIIL&search_mode=CombineSearches) | | #10 AND #2 AND #1 | |  |  |
|  |  |  |  | Databases=SCI-EXPANDED, SSCI, A&HCI, CPCI-S, CPCI-SSH Timespan=All Years | |  |  |
| |  | | --- | | | | | | | | |
| # 26 | | [24](http://apps.isiknowledge.com.myaccess.library.utoronto.ca/summary.do?product=WOS&doc=1&qid=55&SID=4BCl9iO2E7G18kLgIIL&search_mode=CombineSearches) | | #9 AND #2 AND #1 | |  |  |
|  |  |  |  | Databases=SCI-EXPANDED, SSCI, A&HCI, CPCI-S, CPCI-SSH Timespan=All Years | |  |  |
| |  | | --- | | | | | | | | |
| # 25 | | [79](http://apps.isiknowledge.com.myaccess.library.utoronto.ca/summary.do?product=WOS&doc=1&qid=54&SID=4BCl9iO2E7G18kLgIIL&search_mode=CombineSearches) | | #8 AND #2 AND #1 | |  |  |
|  |  |  |  | Databases=SCI-EXPANDED, SSCI, A&HCI, CPCI-S, CPCI-SSH Timespan=All Years | |  |  |
| |  | | --- | | | | | | | | |
| # 24 | | [128](http://apps.isiknowledge.com.myaccess.library.utoronto.ca/summary.do?product=WOS&doc=1&qid=53&SID=4BCl9iO2E7G18kLgIIL&search_mode=CombineSearches) | | #7 AND #2 AND #1 | |  |  |
|  |  |  |  | Databases=SCI-EXPANDED, SSCI, A&HCI, CPCI-S, CPCI-SSH Timespan=All Years | |  |  |
| |  | | --- | | | | | | | | |
| # 23 | | 0 | | #6 AND #2 AND #1 | |  |  |
|  |  |  |  | Databases=SCI-EXPANDED, SSCI, A&HCI, CPCI-S, CPCI-SSH Timespan=All Years | |  |  |
| |  | | --- | | | | | | | | |
| # 22 | | [63](http://apps.isiknowledge.com.myaccess.library.utoronto.ca/summary.do?product=WOS&doc=1&qid=51&SID=4BCl9iO2E7G18kLgIIL&search_mode=CombineSearches) | | #5 AND #2 AND #1 | |  |  |
|  |  |  |  | Databases=SCI-EXPANDED, SSCI, A&HCI, CPCI-S, CPCI-SSH Timespan=All Years | |  |  |
| |  | | --- | | | | | | | | |
| # 21 | | [33](http://apps.isiknowledge.com.myaccess.library.utoronto.ca/summary.do?product=WOS&doc=1&qid=50&SID=4BCl9iO2E7G18kLgIIL&search_mode=CombineSearches) | | #4 AND #2 AND #1 | |  |  |
|  |  |  |  | Databases=SCI-EXPANDED, SSCI, A&HCI, CPCI-S, CPCI-SSH Timespan=All Years | |  |  |
| |  | | --- | | | | | | | | |
| # 20 | | [49](http://apps.isiknowledge.com.myaccess.library.utoronto.ca/summary.do?product=WOS&doc=1&qid=48&SID=4BCl9iO2E7G18kLgIIL&search_mode=CombineSearches) | | #3 AND #2 AND #1 | |  |  |
|  |  |  |  | Databases=SCI-EXPANDED, SSCI, A&HCI, CPCI-S, CPCI-SSH Timespan=All Years | |  |  |
| |  | | --- | | | | | | | | |
| # 19 | | [>100,000](http://apps.isiknowledge.com.myaccess.library.utoronto.ca/summary.do?product=WOS&doc=1&qid=46&SID=4BCl9iO2E7G18kLgIIL&search_mode=CombineSearches) | | #18 OR #17 OR #16 OR #15 OR #14 OR #13 OR #12 OR #11 | |  |  |
|  |  |  |  | Databases=SCI-EXPANDED, SSCI, A&HCI, CPCI-S, CPCI-SSH Timespan=All Years | |  |  |
| |  | | --- | | | | | | | | |
| # 18 | | [6,862](http://apps.isiknowledge.com.myaccess.library.utoronto.ca/summary.do?product=WOS&doc=1&qid=44&SID=4BCl9iO2E7G18kLgIIL&search_mode=GeneralSearch) | | Topic=(((((developing or less* developed or under developed or underdeveloped or middle income or low* income) SAME (economy or economies))))) | |  |  |
|  |  |  |  | Databases=SCI-EXPANDED, SSCI, A&HCI, CPCI-S, CPCI-SSH Timespan=All Years | |  |  |
| |  | | --- | | | | | | | | |
| # 17 | | [1,222](http://apps.isiknowledge.com.myaccess.library.utoronto.ca/summary.do?product=WOS&doc=1&qid=43&SID=4BCl9iO2E7G18kLgIIL&search_mode=GeneralSearch) | | Topic=((transitional countr*)) | |  |  |
|  |  |  |  | Databases=SCI-EXPANDED, SSCI, A&HCI, CPCI-S, CPCI-SSH Timespan=All Years | |  |  |
| |  | | --- | | | | | | | | |
| # 16 | | [17,629](http://apps.isiknowledge.com.myaccess.library.utoronto.ca/summary.do?product=WOS&doc=1&qid=42&SID=4BCl9iO2E7G18kLgIIL&search_mode=GeneralSearch) | | Topic=((lmic or lmics or third world or lami countr*)) | |  |  |
|  |  |  |  | Databases=SCI-EXPANDED, SSCI, A&HCI, CPCI-S, CPCI-SSH Timespan=All Years | |  |  |
| |  | | --- | | | | | | | | |
| # 15 | | [1,393](http://apps.isiknowledge.com.myaccess.library.utoronto.ca/summary.do?product=WOS&doc=1&qid=41&SID=4BCl9iO2E7G18kLgIIL&search_mode=GeneralSearch) | | Topic=((low SAME middle SAME countr*)) | |  |  |
|  |  |  |  | Databases=SCI-EXPANDED, SSCI, A&HCI, CPCI-S, CPCI-SSH Timespan=All Years | |  |  |
| |  | | --- | | | | | | | | |
| # 14 | | [1,642](http://apps.isiknowledge.com.myaccess.library.utoronto.ca/summary.do?product=WOS&doc=1&qid=39&SID=4BCl9iO2E7G18kLgIIL&search_mode=GeneralSearch) | | Topic=(low SAME middle countr*) | |  |  |
|  |  |  |  | Databases=SCI-EXPANDED, SSCI, A&HCI, CPCI-S, CPCI-SSH Timespan=All Years | |  |  |
| |  | | --- | | | | | | | | |
| # 13 | | 0 | | Topic=((low SAME middle adj3 countr*)) | |  |  |
|  |  |  |  | Databases=SCI-EXPANDED, SSCI, A&HCI, CPCI-S, CPCI-SSH Timespan=All Years | |  |  |
| |  | | --- | | | | | | | | |
| # 12 | | [2,341](http://apps.isiknowledge.com.myaccess.library.utoronto.ca/summary.do?product=WOS&doc=1&qid=37&SID=4BCl9iO2E7G18kLgIIL&search_mode=GeneralSearch) | | Topic=((((developing or less* developed or under developed or underdeveloped or middle income or low* income) adj (economy or economies)))) | |  |  |
|  |  |  |  | Databases=SCI-EXPANDED, SSCI, A&HCI, CPCI-S, CPCI-SSH Timespan=All Years | |  |  |
| |  | | --- | | | | | | | | |
| # 11 | | [>100,000](http://apps.isiknowledge.com.myaccess.library.utoronto.ca/summary.do?product=WOS&doc=1&qid=36&SID=4BCl9iO2E7G18kLgIIL&search_mode=GeneralSearch) | | Topic=((((developing or less* developed or under developed or underdeveloped or middle income or low* income or underserved or under served or deprived or poor*) SAME (countr* or nation? or population? or world)))) | |  |  |
|  |  |  |  | Databases=SCI-EXPANDED, SSCI, A&HCI, CPCI-S, CPCI-SSH Timespan=All Years | |  |  |
| |  | | --- | | | | | | | | |
| # 10 | | [>100,000](http://apps.isiknowledge.com.myaccess.library.utoronto.ca/summary.do?product=WOS&doc=1&qid=35&SID=4BCl9iO2E7G18kLgIIL&search_mode=GeneralSearch) | | Topic=(((Africa or Asia or Caribbean or West Indies or South America or Latin America or Central America))) | |  |  |
|  |  |  |  | Databases=SCI-EXPANDED, SSCI, A&HCI, CPCI-S, CPCI-SSH Timespan=All Years | |  |  |
| |  | | --- | | | | | | | | |
| # 9 | | [28,192](http://apps.isiknowledge.com.myaccess.library.utoronto.ca/summary.do?product=WOS&doc=1&qid=34&SID=4BCl9iO2E7G18kLgIIL&search_mode=GeneralSearch) | | Topic=(((Azerbaijan or Georgia or Georgian Soviet Socialist Republic or Kosovo or Moldova or Moldavian Soviet Socialist Republic or Moldovan Soviet Socialist Republic or Turkmenistan or Turkmen Soviet Socialist Republic or Ukraine or Ukrainian National Republic or Ukrainian State or Ukrainian Soviet Socialist Republic))) | |  |  |
|  |  |  |  | Databases=SCI-EXPANDED, SSCI, A&HCI, CPCI-S, CPCI-SSH Timespan=All Years | |  |  |
| |  | | --- | | | | | | | | |
| # 8 | | [>100,000](http://apps.isiknowledge.com.myaccess.library.utoronto.ca/summary.do?product=WOS&doc=1&qid=33&SID=4BCl9iO2E7G18kLgIIL&search_mode=GeneralSearch) | | Topic=(((Sudan or Swaziland or China or PRC or Indonesia or Netherlands East Indies or Dutch East Indies or Kiribati or Gilbert Islands or Marshall islands or rmi or Trust Territory of the Pacific Islands or Micronesia or Trust Territory of the Pacific Islands or Ponape or Truk or yap Districts or fsm or Mongolia or Papua New Guinea or png or Papua or New Guinea or Philippines or Samoa or Solomon Islands or Thailand or Siam or Timor-Leste or East Timor or Portuguese Timor or Tonga or Friendly Islands or Vanuatu or New Hebrides or Albania or armenia*))) | |  |  |
|  |  |  |  | Databases=SCI-EXPANDED, SSCI, A&HCI, CPCI-S, CPCI-SSH Timespan=All Years | |  |  |
| |  | | --- | | | | | | | | |
| # 7 | | [>100,000](http://apps.isiknowledge.com.myaccess.library.utoronto.ca/summary.do?product=WOS&doc=1&qid=32&SID=4BCl9iO2E7G18kLgIIL&search_mode=GeneralSearch) | | Topic=(((Belize or British Honduras or Bolivia or Ecuador or El Salvador or Guatemala or Guyana or British Guiana or Honduras or Nicaragua or Paraguay or Djibouti or Afars or Issas or French Somaliland or Egypt or United Arab Republic or Iran or Persia or Iraq or Jordan or Transjordan or Morocco or Syrian Arab Republic or Syria or Tunisia or Tunisian Republic or est Bank or Gaza or Bhutan or India or Maldives or Pakistan or Sri Lanka or Serendib or Ceylon))) | |  |  |
|  |  |  |  | Databases=SCI-EXPANDED, SSCI, A&HCI, CPCI-S, CPCI-SSH Timespan=All Years | |  |  |
| |  | | --- | | | | | | | | |
| # 6 | | [114](http://apps.isiknowledge.com.myaccess.library.utoronto.ca/summary.do?product=WOS&doc=1&qid=31&SID=4BCl9iO2E7G18kLgIIL&search_mode=GeneralSearch) | | Topic=("Sao Tome and Principe") | |  |  |
|  |  |  |  | Databases=SCI-EXPANDED, SSCI, A&HCI, CPCI-S, CPCI-SSH Timespan=All Years | |  |  |
| |  | | --- | | | | | | | | |
| # 5 | | [>100,000](http://apps.isiknowledge.com.myaccess.library.utoronto.ca/summary.do?product=WOS&doc=1&qid=30&SID=4BCl9iO2E7G18kLgIIL&search_mode=GeneralSearch) | | Topic=(((Cambodia or Khmer Republic or Kampuchea or Democratic People's Republic of Korea or north korea or DPRK or Lao PDR or Lao People's Democratic Republic or Laos or Myanmar or Myanma or burma or Vietnam or SRV or Kyrgyz Republic or Kyrgyzstan or Kirghiz Soviet Socialist Republic or Tajikistan or Tajik Soviet Socialist Republic or Uzbekistan or Uzbek Soviet Socialist Republic or Haiti or Yemen or Afghanistan or Bangladesh or East Bengal or East Pakistan or Nepal or Angola or Cameroon or Republic of Congo or Cote d'Ivoire or Ivory Coast or Lesotho or Basutoland or Nigeria))) | |  |  |
|  |  |  |  | Databases=SCI-EXPANDED, SSCI, A&HCI, CPCI-S, CPCI-SSH Timespan=All Years | |  |  |
| |  | | --- | | | | | | | | |
| # 4 | | [51,593](http://apps.isiknowledge.com.myaccess.library.utoronto.ca/summary.do?product=WOS&doc=1&qid=29&SID=4BCl9iO2E7G18kLgIIL&search_mode=GeneralSearch) | | Topic=(((rwanda OR ruanda OR german east africa OR senegal OR senegambia OR mali federation OR sierra leone OR somalia OR somali OR tanzania OR united republic of tanganyika OR zanzibar OR togo OR togolese republic OR togoland OR uganda OR zambia OR northern rhodesia OR zimbabwe OR rhodesia))) | |  |  |
|  |  |  |  | Databases=SCI-EXPANDED, SSCI, A&HCI, CPCI-S, CPCI-SSH Timespan=All Years | |  |  |
| |  | | --- | | | | | | | | |
| # 3 | | [>100,000](http://apps.isiknowledge.com.myaccess.library.utoronto.ca/summary.do?product=WOS&doc=1&qid=28&SID=4BCl9iO2E7G18kLgIIL&search_mode=GeneralSearch) | | Topic=(((benin OR burkina faso OR fasso OR upper valto OR burundi OR urundi OR central african republic OR ubangi shari OR central african empire OR chad OR comoros OR comores OR congo OR zaire OR eritrea OR ethiopia OR abyssinia OR italian east africa OR fdre OR gambia OR ghana OR gold coast OR guinea OR kenya OR british east africa OR liberia OR madagascar OR malagasy republic OR malawi OR british central african protectorate OR nyasaland OR mali OR french sudan OR sudanese republic OR mauritania mozambique OR portuguese east africa OR niger))) | |  |  |
|  |  |  |  | Databases=SCI-EXPANDED, SSCI, A&HCI, CPCI-S, CPCI-SSH Timespan=All Years | |  |  |
| |  | | --- | | | | | | | | |
| # 2 | [>100,000](http://apps.isiknowledge.com.myaccess.library.utoronto.ca/summary.do?product=WOS&doc=1&qid=27&SID=4BCl9iO2E7G18kLgIIL&search_mode=GeneralSearch) | | Topic=(((learn* or instruc* or train* or educat* or course* or workshop* or work-shop* or program* or teach*))) | |  | |  |
|  |  |  | Databases=SCI-EXPANDED, SSCI, A&HCI, CPCI-S, CPCI-SSH Timespan=All Years | |  |  |  |
| |  | | --- | | | | | | | | |
| # 1 | | [24,554](http://apps.isiknowledge.com.myaccess.library.utoronto.ca/summary.do?product=WOS&doc=1&qid=26&SID=4BCl9iO2E7G18kLgIIL&search_mode=GeneralSearch) | | Topic=(((emergency medicine) OR (disaster medicine) or (emergency medical service*) OR (emergenc* SAME medic*) OR (disast* SAME medic*) or (emergenc* SAME residenc*))) | |  | Bottom of Form |
|  |  |  |  | Databases=SCI-EXPANDED, SSCI, A&HCI, CPCI-S, CPCI-SSH Timespan=All Years | |  |  |
